# Infants Admitted to US Intensive Care Units for RSV Infection During the 2022 Seasonal Peak

**DOI:** 10.1001/jamanetworkopen.2023.28950

**Published:** 2023-08-15

**Authors:** Natasha Halasa, Laura D. Zambrano, Justin Z. Amarin, Laura S. Stewart, Margaret M. Newhams, Emily R. Levy, Steven L. Shein, Christopher L. Carroll, Julie C. Fitzgerald, Marian G. Michaels, Katherine Bline, Melissa L. Cullimore, Laura Loftis, Vicki L. Montgomery, Asumthia S. Jeyapalan, Pia S. Pannaraj, Adam J. Schwarz, Natalie Z. Cvijanovich, Matt S. Zinter, Aline B. Maddux, Melania M. Bembea, Katherine Irby, Danielle M. Zerr, Joseph D. Kuebler, Christopher J. Babbitt, Mary Glas Gaspers, Ryan A. Nofziger, Michele Kong, Bria M. Coates, Jennifer E. Schuster, Shira J. Gertz, Elizabeth H. Mack, Benjamin R. White, Helen Harvey, Charlotte V. Hobbs, Heda Dapul, Andrew D. Butler, Tamara T. Bradford, Courtney M. Rowan, Kari Wellnitz, Mary Allen Staat, Cassyanne L. Aguiar, Saul R. Hymes, Adrienne G. Randolph, Angela P. Campbell

**Affiliations:** 1 Division of Pediatric Infectious Diseases, Department of Pediatrics, Vanderbilt University Medical Center, Nashville, Tennessee; 2 Coronavirus and Other Respiratory Viruses Division, Centers for Disease Control and Prevention, Atlanta, Georgia; 3 Department of Anesthesiology, Critical Care, and Pain Medicine, Boston Children’s Hospital, Boston, Massachusetts; 4 Divisions of Pediatric Infectious Diseases and Pediatric Critical Care Medicine, Department of Pediatric and Adolescent Medicine, Mayo Clinic, Rochester, Minnesota; 5 Division of Pediatric Critical Care Medicine, Rainbow Babies and Children’s Hospital, Cleveland, Ohio; 6 Department of Pediatrics, Connecticut Children’s Medical Center, Hartford; 7 Department of Anesthesiology and Critical Care, Children’s Hospital of Philadelphia, University of Pennsylvania Perelman School of Medicine, Philadelphia; 8 Division of Infectious Diseases, Department of Pediatrics, UPMC Children’s Hospital of Pittsburgh, Pittsburgh, Pennsylvania; 9 Division of Pediatric Critical Care Medicine, Nationwide Children’s Hospital, Columbus, Ohio; 10 Division of Pediatric Critical Care, Department of Pediatrics, Children’s Hospital and Medical Center, Omaha, Nebraska; 11 Section of Critical Care Medicine, Department of Pediatrics, Texas Children’s Hospital, Houston; 12 Department of Pediatrics, University of Louisville and Norton Children’s Hospital, Louisville, Kentucky; 13 Division of Pediatric Critical Care Medicine, University of Miami Miller School of Medicine, Miami, Florida; 14 Division of Infectious Diseases, Children’s Hospital Los Angeles and Departments of Pediatrics and Molecular Microbiology and Immunology, University of Southern California, Los Angeles; 15 Division of Critical Care Medicine, Children’s Hospital Orange County, Orange, California; 16 Division of Critical Care, Department of Pediatrics, University of California, San Francisco Benioff Children’s Hospital Oakland, Oakland; 17 Division of Critical Care, Department of Pediatrics, University of California, San Francisco Benioff Children’s Hospital San Francisco, San Francisco; 18 Department of Pediatrics, Section of Critical Care Medicine, University of Colorado School of Medicine and Children’s Hospital Colorado, Aurora; 19 Department of Anesthesiology and Critical Care Medicine, Johns Hopkins University School of Medicine, Baltimore, Maryland; 20 Section of Pediatric Critical Care, Department of Pediatrics, Arkansas Children’s Hospital, Little Rock; 21 Division of Pediatric Infectious Diseases, Department of Pediatrics, Seattle Children’s Hospital, Seattle, Washington; 22 Division of Pediatric Critical Care, Department of Pediatrics, Golisano Children’s Hospital, University of Rochester Medical Center, Rochester, New York; 23 Division of Pediatric Critical Care, Miller Children’s and Women’s Hospital of Long Beach, Long Beach, California; 24 Division of Critical Care, Department of Pediatrics, Banner Children’s at Diamond Children’s Medical Center, Tucson, Arizona; 25 Division of Critical Care Medicine, Akron Children’s Hospital, Akron, Ohio; 26 Division of Pediatric Critical Care Medicine, Department of Pediatrics, University of Alabama at Birmingham; 27 Division of Pediatric Critical Care Medicine, Ann and Robert H. Lurie Children’s Hospital of Chicago, Northwestern University Feinberg School of Medicine, Chicago, Illinois; 28 Division of Pediatric Infectious Diseases, Department of Pediatrics, Children’s Mercy Kansas City, Kansas City, Missouri; 29 Division of Pediatric Critical Care, Department of Pediatrics, Cooperman Barnabas Medical Center, Livingston, New Jersey; 30 Division of Pediatric Critical Care Medicine, Medical University of South Carolina, Charleston; 31 Division of Pediatric Critical Care, Department of Pediatrics, University of Utah, Salt Lake City; 32 Division of Pediatric Critical Care, Rady Children’s Hospital-San Diego, San Diego, California; 33 Division of Infectious Diseases, Department of Pediatrics, University of Mississippi Medical Center, Jackson; 34 Division of Pediatric Critical Care Medicine, Department of Pediatrics, New York University Grossman School of Medicine, New York; 35 Division of Pediatric Critical Care, St Christopher’s Hospital for Children, Philadelphia, Pennsylvania; 36 Division of Cardiology, Department of Pediatrics, Louisiana State University Health Sciences Center and Children’s Hospital of New Orleans, New Orleans; 37 Division of Pediatric Critical Care Medicine, Department of Pediatrics, Indiana University School of Medicine, Riley Hospital for Children, Indianapolis; 38 Division of Pediatric Critical Care, Stead Family Department of Pediatrics, University of Iowa Carver College of Medicine, Iowa City; 39 Department of Pediatrics, University of Cincinnati College of Medicine, Cincinnati Children’s Hospital Medical Center, Cincinnati, Ohio; 40 Division of Pediatric Rheumatology, Children’s Hospital of The King’s Daughters, Eastern Virginia Medical School, Norfolk; 41 Division of Pediatric Infectious Diseases, Department of Pediatrics, Bernard and Millie Duker Children’s Hospital, Albany Med Health System, Albany, New York; 42 Department of Pediatrics, Harvard Medical School, Boston, Massachusetts; 43 Department of Anaesthesia, Harvard Medical School, Boston, Massachusetts

## Abstract

**Importance:**

Respiratory syncytial virus (RSV) is the leading cause of lower respiratory tract infections (LRTIs) and infant hospitalization worldwide.

**Objective:**

To evaluate the characteristics and outcomes of RSV-related critical illness in US infants during peak 2022 RSV transmission.

**Design, Setting, and Participants:**

This cross-sectional study used a public health prospective surveillance registry in 39 pediatric hospitals across 27 US states. Participants were infants admitted for 24 or more hours between October 17 and December 16, 2022, to a unit providing intensive care due to laboratory-confirmed RSV infection.

**Exposure:**

Respiratory syncytial virus.

**Main Outcomes and Measures:**

Data were captured on demographics, clinical characteristics, signs and symptoms, laboratory values, severity measures, and clinical outcomes, including receipt of noninvasive respiratory support, invasive mechanical ventilation, vasopressors or extracorporeal membrane oxygenation, and death. Mixed-effects multivariable log-binomial regression models were used to assess associations between intubation status and demographic factors, gestational age, and underlying conditions, including hospital as a random effect to account for between-site heterogeneity.

**Results:**

The first 15 to 20 consecutive eligible infants from each site were included for a target sample size of 600. Among the 600 infants, the median (IQR) age was 2.6 (1.4-6.0) months; 361 (60.2%) were male, 169 (28.9%) were born prematurely, and 487 (81.2%) had no underlying medical conditions. Primary reasons for admission included LRTI (594 infants [99.0%]) and apnea or bradycardia (77 infants [12.8%]). Overall, 143 infants (23.8%) received invasive mechanical ventilation (median [IQR], 6.0 [4.0-10.0] days). The highest level of respiratory support for nonintubated infants was high-flow nasal cannula (243 infants [40.5%]), followed by bilevel positive airway pressure (150 infants [25.0%]) and continuous positive airway pressure (52 infants [8.7%]). Infants younger than 3 months, those born prematurely (gestational age <37 weeks), or those publicly insured were at higher risk for intubation. Four infants (0.7%) received extracorporeal membrane oxygenation, and 2 died. The median (IQR) length of hospitalization for survivors was 5 (4-10) days.

**Conclusions and Relevance:**

In this cross-sectional study, most US infants who required intensive care for RSV LRTIs were young, healthy, and born at term. These findings highlight the need for RSV preventive interventions targeting all infants to reduce the burden of severe RSV illness.

## Key Points

**Question:**

What were the clinical characteristics and outcomes of respiratory syncytial virus (RSV)–related critical illness in US infants during peak 2022 RSV transmission?

**Findings:**

This cross-sectional surveillance study of 600 infants across 39 hospitals requiring intensive care for RSV infection found that most were delivered full-term and previously healthy. Infants aged less than 3 months and those born prematurely were at higher risk for intubation.

**Meaning:**

These findings support the use of new preventative interventions, including long-lasting monoclonal antibodies in all infants and maternal vaccination.

## Teaser

This cross-sectional study evaluates the characteristics of infants admitted to US intensive care units for respiratory syncytial virus (RSV) infection during the 2022 seasonal peak.

## Introduction

Respiratory syncytial virus (RSV) is the leading cause of respiratory-related hospitalizations in young children worldwide.^1,2^ In the US, RSV annually accounts for approximately 57 000 hospitalizations in children younger than 5 years^3^ and is the leading cause of hospitalizations in the first year of life^4^; approximately 1 in 5 RSV-positive hospitalized young children were admitted to the intensive care unit (ICU).^5,6^ Although most children hospitalized with RSV are previously healthy and born at term,^3,5,7,8^ children with a history of prematurity or certain underlying medical conditions such as congenital heart disease, neurologic or neurodevelopmental disorders, chronic lung disease, and immunocompromising conditions are at higher risk for life-threatening RSV disease.^9^

Palivizumab, a monoclonal antibody (IgG) targeting the RSV F protein, is given monthly by intramuscular injection during RSV season to prevent RSV-associated lower respiratory tract infection (LRTI). It has previously been the only US-licensed product for this purpose, and its use is limited to high-risk infants.^10^ However, the significant cost of palivizumab is a barrier to its expanded use globally. The US Food and Drug Administration has just approved a long-acting (approximately 150 days) monoclonal RSV-neutralizing antibody and a maternal vaccine for RSV prevention is under consideration.^11^ These products may protect both high-risk and healthy infants from medically attended RSV-associated LRTI.^12,13,14,15,16^ Identifying which infants are at risk for severe RSV disease is essential for assessing future clinical effectiveness and guiding product usage recommendations.

The COVID-19 pandemic disrupted typical RSV circulation patterns, leading to atypical US epidemics during 2021 and 2022.^17,18^ Specifically, a surge in RSV-related hospitalizations and ICU admissions among infants and young children occurred in the fall of 2022.^19^ Therefore, we aimed to describe the characteristics, clinical course, and outcomes, including life-threatening complications, of these US infants admitted for pediatric intensive care during this period.

## Methods

### Study Design

This investigation included infants (<1 year old) from 39 US pediatric hospitals representing 27 states admitted over 2 months between October 17 and December 16, 2022, coinciding with the peak of the US RSV season.^19^ We selected October 17 as the study start date because most participating sites had reported RSV activity by that date, which reflected national surveillance data.^20^ Most sites participating in the RSV Pediatric Intensive Care (RSV-PIC) registry had previously participated in US Centers for Disease Control and Prevention (CDC)–funded pediatric critical illness studies,^21^ and others were recruited to ensure geographic representation. Inclusion in the RSV-PIC registry required admission to the ICU or high acuity unit for 24 or more hours for RSV-related illness, symptom onset of less than 10 days before hospitalization, and evidence of laboratory-confirmed RSV before or within 72 hours after hospitalization. Infants previously included in the RSV-PIC registry and newborns never discharged after birth were excluded. To ensure geographic representation, we included the first 15 to 20 consecutive eligible infants from each participating site. Clinical characteristics, interventions, laboratory tests (including other respiratory viral testing up to 72 hours and bacterial testing up to 3 days of hospital admission), and outcomes were collected using a standardized data collection form. Three physician investigators (N.B.H., E.R.L., and A.P.C.) adjudicated bacterial coinfection.

### Ethics

The Boston Children’s Hospital institutional review board (IRB) reviewed and approved the protocol. Thirty-one sites relied on Boston Children’s Hospital’s single IRB; 7 had site-specific IRB approval. A waiver of consent was granted by all respective IRBs because this was considered a minimal risk study because the data collection was through medical record reviews and included limited protected health information. This activity was reviewed by CDC and determined to meet the requirements of public health surveillance per 45 CFR §46.101(b)(4). This report adheres to the Strengthening the Reporting of Observational Studies in Epidemiology (STROBE) reporting guideline for cross-sectional studies.^22^

### Variables of Interest

We assessed demographic and clinical characteristics, including age, sex, race and Hispanic ethnicity, site region, underlying medical conditions, and presence of pulmonary infiltrates on chest radiograph per radiology report. Data on race and ethnicity were collected from electronic health records. Prior studies have observed racial and ethnic disparities in the severity of life-threatening RSV disease. Therefore, these data enabled the assessment of potential disparities in RSV severity, as indicated by intubation status, across different racial and ethnic groups. Infants were considered previously healthy if they had no underlying medical conditions (defined in eAppendix in Supplement 1) and were not receiving prescription medications for chronic conditions.^23^

We also included laboratory markers of inflammation and disease severity, including complete blood count and blood gas values, signs and symptoms, and clinical outcomes.^23^ To assess illness severity, we used diagnosis of pediatric acute respiratory distress syndrome (PARDS; defined in eAppendix in Supplement 1),^24^ the pediatric sequential organ failure assessment (pSOFA) score,^25^ and the pediatric logistic organ dysfunction-2 (PELOD-2) score.^26,27^ We captured data on receipt of high flow nasal cannula, noninvasive or invasive mechanical ventilation, vasopressors, or extracorporeal membrane oxygenation (ECMO), and death during the index hospitalization.

### Statistical Analysis

Demographic characteristics, gestational age, indication for admission, and presence of underlying conditions were stratified by tracheal intubation with receipt of invasive mechanical ventilation (intubation) status. Clinical presentation (including interventions received within the first 24 hours of hospitalization) and overall clinical course and outcomes were described by age group (0-2, 3-5, and 6-11 months). Categorical variables were analyzed by Pearson χ^2^ test or Fisher exact test and Cochran-Armitage tests for trend for comparisons by intubation status and age group. Continuous variables were analyzed by intubation status through Kruskal-Wallis or age group through Jonckheere-Terpstra tests for trend. Two-sided *P* values less than .05 were considered statistically significant.

Associations between intubation status and demographic factors, gestational age, and underlying conditions were evaluated through mixed-effects multivariable log-binomial regression models to calculate prevalence ratios (appropriate for cross-sectional data), including hospital as a random effect to account for between-site heterogeneity. Where models did not converge given limited sample size, exact Poisson regression models were used. Variables associated with intubation status (α < .35) were considered potential covariates in full multivariable models if they could plausibly be associated with each exposure of interest and intubation status. Variables were retained in multivariable models if removal altered the full model effect estimate by 10% or more. Data analysis was performed in SAS version 9.4 (SAS Institute) and R version 4.2.2 (R Project for Statistical Computing).

## Results

### Demographic and Clinical Characteristics

Of the 600 infants in the RSV-PIC registry, 559 (93.2%) were admitted within the first month of the study period, at which time two-thirds of sites had met their enrollment target. The median (IQR) age was 2.6 (1.4-6.0) months, and 361 were male (60%); 487 infants (81%) were previously healthy, 169 (29%) were born prematurely, and nearly one-quarter were intubated (Table 1). The eFigure in Supplement 1 displays the age distribution by months, stratified by intubation status. Compared with nonintubated infants, intubated infants were younger, were more frequently delivered prematurely, and more frequently had public insurance (Table 1). Although the proportions of infants included from each census region were similar, intubation status varied by census region with more intubated infants admitted in the Midwest (53 infants [37.1%]) and South (42 infants [29.4%]). Underlying medical conditions were not higher in the intubated group, but there was a lower frequency of nonrespiratory/noncardiac disorders (Table 1 and eTable 1 in Supplement 1).

**Table 1.** Demographic and Clinical Characteristics of Infants Admitted to the Intensive Care or High Acuity Unit With Respiratory Syncytial Virus Infection

Characteristic	Infants, No. (%)			*P* value^a^
	All (N = 600)	Nonintubated (n = 457)	Intubated (n = 143)	
Age, median (IQR), mo	2.6 (1.4-6.0)	3.1 (1.6-6.4)	1.9 (1.0-3.2)	<.001
Age group				
0-2 mos	323 (53.8)	222 (48.6)	101 (70.6)	<.001
3-5 mos	127 (21.2)	106 (23.2)	21 (14.7)	
6-11 mos	150 (25.0)	129 (28.2)	21 (14.7)	
Sex				
Male	361 (60.2)	277 (60.6)	84 (58.7)	.69
Female	239 (39.8)	180 (39.4)	59 (41.3)	
Race and ethnicity				
Hispanic	135 (22.5)	102 (22.3)	33 (23.1)	NA
Non-Hispanic Asian	13 (2.2)	11 (2.4)	2 (1.4)	
Non-Hispanic Black	95 (15.8)	75 (16.4)	20 (14.0)	
Non-Hispanic White	265 (44.2)	199 (43.5)	66 (46.2)	
Multiple or other^b^	30 (5.0)	21 (4.6)	9 (6.3)	
Unknown	62 (10.3)	49 (10.7)	13 (9.1)	
SVI score, median (IQR)^c^	0.50 (0.39-0.64)	0.50 (0.40-0.64)	0.50 (0.38-0.65)	.98
Insurance				
Public	336 (56.0)	242 (53.0)	94 (65.7)	.050
Private	240 (40.0)	196 (42.9)	44 (30.8)	
Self-pay	11 (1.8)	9 (2.0)	2 (1.4)	
Other or unknown	13 (2.2)	10 (2.2)	3 (2.1)	
Prematurity^d^	169 (28.9)	116 (26.1)	53 (37.5)	.01
Gestational age, median (IQR)^e^	34.0 (32.0-35.7)	34.0 (32.4-35.7)	34.0 (32.0-35.4)	.91
Multiple pregnancy^f^	28 (4.7)	16 (3.5)	12 (8.4)	.18
Underlying conditions				
None	487 (81.2)	364 (79.6)	123 (86.0)	.07
At least one	113 (18.8)	93 (20.4)	20 (14.0)	
Nonrespiratory, noncardiac	48 (8.0)	46 (10.1)	2 (1.4)	<.001
Cardiac, nonrespiratory	20 (3.3)	15 (3.3)	5 (3.5)	.90
Respiratory	45 (7.5)	32 (7.0)	13 (9.1)	.41
Chronic lung disease	22 (3.7)	14 (3.1)	8 (5.6)	.16
Neurologic	13 (2.2)	9 (2.0)	4 (2.8)	.52
Trisomy 21	8 (1.3)	8 (1.8)	0	.21
Reason for admission				
LRTI	594 (99.0)	453 (99.1)	141 (98.6)	.58
Apnea or bradycardia	77 (12.8)	36 (7.9)	41 (28.7)	<.001
Cardiac arrest at home with CPR	3 (0.5)	1 (0.2)	2 (1.4)	.14
CNS infection	2 (0.3)	0	2 (1.4)	.06
Shock requiring vasopressors	5 (0.8)	0	5 (3.5)	.001
Site region				
Northeast	146 (24.3)	125 (27.4)	21 (14.7)	<.001
Midwest	145 (24.2)	92 (20.1)	53 (37.1)	
South	154 (25.7)	112 (24.5)	42 (29.4)	
West	155 (25.8)	128 (28.0)	27 (18.9)	

Abbreviations: CNS, central nervous system; CPR, cardiopulmonary resuscitation; LRTI, lower respiratory tract infection; NA, not applicable; SVI, social vulnerability index.

^a^ Fisher exact test performed if any expected cell count was less than 5.

^b^ Multiple or other race and ethnicity group included infants who were of multiple races or were classified as Native Hawaiian/Pacific Islander or American Indian/Alaskan Native.

^c^ A total of 598 observations.

^d^ Denominator was for infants with documented prematurity status (total of 585 observations).

^e^ Gestational age provided for infants born at less than 37 weeks’ gestation.

^f^ Multiple pregnancy status was collected only for 320 infants aged less than 90 days.

**Figure 1.**
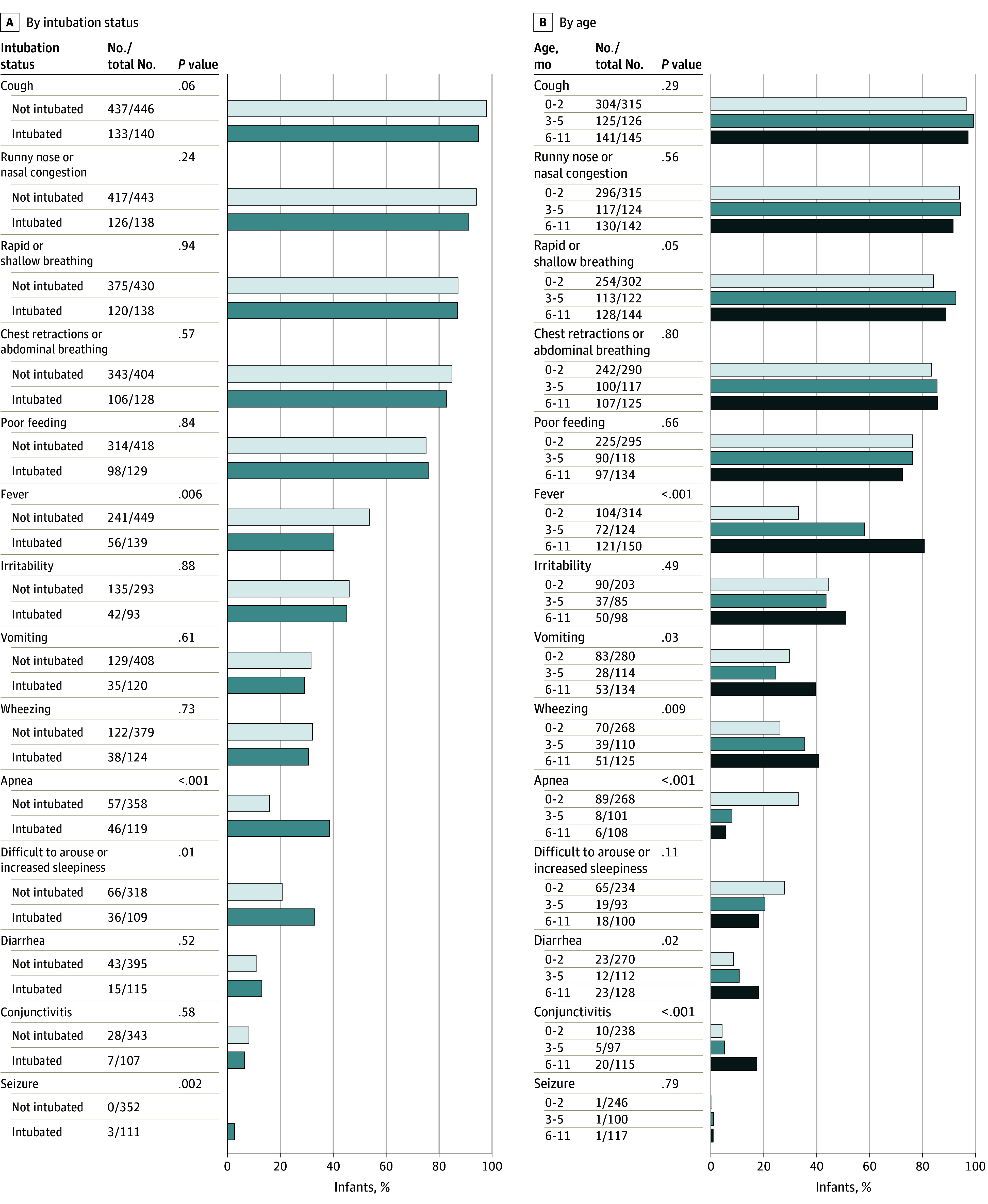
Signs and Symptoms in Infants Requiring Intensive Care for Respiratory Syncytial Virus Infection

The primary reason for admission among most infants was LRTI (594 infants [99%]), but infants who were intubated had a higher frequency of apnea or bradycardia (77 infants [13%]) and were more frequently younger than 3 months (Table 1). Intubated infants had a higher frequency of apnea, seizures, and increased sleepiness (difficult to arouse) but lower frequency of fever (Figure 1A). Older infants had higher frequency of fever, wheezing, rapid or shallow breathing, conjunctivitis, vomiting, and diarrhea, while younger infants had higher frequency of apnea (Figure 1B).

### Clinical Presentation and Hospital Course

Among the 600 infants, 572 (95.3%) required oxygen support at admission (Table 2). Overall, 143 infants (24%) received invasive mechanical ventilation (median [IQR], 6.0 [4.0-10] days). Among those intubated, 101 infants (70.6%) were younger than 3 months. The highest level of respiratory support for nonintubated infants was high-flow nasal cannula (HFNC; 243 infants [40.5%]), followed by bilevel positive airway pressure (BiPAP; 150 infants [25.0%]) and continuous positive airway pressure (CPAP; 52 infants [8.7%]). The median (IQR) length of hospitalization for survivors was 5 (4-10) days. HFNC was the most common respiratory support at admission across all age groups. For infants younger than 3 months, intubation was the second most common respiratory support and for infants aged 3 months and older it was BiPAP or noninvasive ventilation. These patterns remained consistent throughout the hospital course; although the frequency of intubation in all age groups increased, the proportion remained highest for infants younger than 3 months. The median (IQR) days of invasive mechanical ventilation did not differ significantly between age groups (6 [4-10] days).

**Table 2.** Clinical Presentation and Course of Infants Admitted to the Intensive Care or High Acuity Unit With Respiratory Syncytial Virus Infection Stratified by Age Group in Months

Clinical presentation and course	Infants, No. (%)				*P* value^a^
	Total (N = 600)	0-2 mos (n = 323)	3-5 mos (n = 127)	6-11 mos (n = 150)	
Reason for admission					
LRTI	594 (99.0)	317 (98.1)	127 (100.0)	150 (100.0)	.04
Apnea or bradycardia	77 (12.8)	67 (20.7)	6 (4.7)	4 (2.7)	<.001
Cardiac arrest at home with CPR	3 (0.5)	3 (0.9)	0	0	.14
CNS infection	2 (0.3)	2 (0.6)	0	0	.23
Shock requiring vasopressors	5 (0.8)	3 (0.9)	1 (0.8)	1 (0.7)	.75
Interventions within first 24 h					
Highest level of respiratory support^b^					
None	28 (4.7)	16 (5.0)	4 (3.1)	8 (5.3)	.71
Low-flow supplemental oxygen	51 (8.5)	30 (9.3)	6 (4.7)	15 (10.0)	.96
High-flow nasal cannula oxygen	252 (42.0)	116 (35.9)	71 (55.9)	65 (43.3)	.03
Continuous positive airway pressure	52 (8.7)	34 (10.5)	7 (5.5)	11 (7.3)	.18
BiPAP/NIV	114 (19.0)	52 (16.1)	24 (18.9)	38 (25.3)	.02
Invasive mechanical ventilation	103 (17.2)	75 (23.2)	15 (11.8)	13 (8.7)	<.001
Cardiovascular support	10 (1.7)	6 (1.9)	2 (1.6)	2 (1.3)	.67
Vasopressors	6 (1.0)	3 (0.9)	1 (0.8)	2 (1.3)	.72
Clinical course					
Highest level of respiratory support					
None	1 (0.2)	1 (0.3)	0	0	>.99
Low-flow supplemental oxygen	11 (1.8)	5 (1.5)	1 (0.8)	5 (3.3)	.30
High-flow nasal cannula oxygen	243 (40.5)	114 (35.3)	67 (52.8)	62 (41.3)	.07
Continuous positive airway pressure	52 (8.7)	33 (10.2)	7 (5.5)	12 (8.0)	.30
BiPAP/NIV	150 (25.0)	69 (21.4)	31 (24.4)	50 (33.3)	.01
Invasive mechanical ventilation	143 (23.8)	101 (31.3)	21 (16.5)	21 (14.0)	<.001
Days ventilated, median (IQR)	6 (4-10)	6 (4-9)	5 (3-10)	7 (5-11)	.59
Vasopressor-dependent shock	27 (4.5)	19 (5.9)	3 (2.4)	5 (3.3)	.15
ECMO	4 (0.7)	2 (0.6)	0	2 (1.3)	.53
Days in ICU, median (IQR)^c^	3 (2-7)	4 (2-8)	3 (2-5)	3 (2-6)	.002
Days in hospital, median (IQR)^c^	5 (4-10)	6 (4-12)	5 (3-7)	5 (3-8)	.001
In-hospital death	2 (0.3)	0	0	2 (1.3)	.11
Severity scores					
pSOFA score, median (IQR)	4 (3-4)	4 (3-5)	3 (3-4)	3 (3-4)	<.001
PELOD-2 score, median (IQR)	2 (1-4)	2 (2-5)	2 (0-4)	0 (0-2)	<.001
PARDS criteria met	120 (20.0)	72 (22.3)	21 (16.5)	27 (18.0)	.19

Abbreviations: BiPAP, bilevel positive airway pressure; CNS, central nervous system; CPR, cardiopulmonary resuscitation; ECMO, extracorporeal membrane oxygenation; ICU, intensive care unit; LRTI, lower respiratory tract infection; NIV, noninvasive ventilation; PARDS, pediatric acute respiratory distress syndrome; PELOD, pediatric logistic organ dysfunction; pSOFA, pediatric sequential organ failure assessment.

^a^ Fisher exact test performed if any expected cell count was less than 5.

^b^ Data displayed reflect responses to a question as to whether the infant received mechanical ventilation and/or oxygen support during the first 24 hours of hospital admission. Low-flow supplemental oxygen and invasive mechanical ventilation were also noted at admission if reported on day 1 of daily clinical variables. This information may reflect pre–pediatric intensive care unit care.

^c^ A total of 598 survivors.

Infants younger than 3 months had higher disease severity scores (ie, pSOFA and PELOD-2) and longer duration of ICU and hospital stay compared with the other age groups (Table 2). Four infants required ECMO; 2 infants died, 1 of whom was on ECMO. Details of severe clinical outcomes are provided in eTable 2 in Supplement 1.

### Palivizumab Use

For 15 of the 17 infants born at less than 29 weeks’ gestation there was no documentation of receiving palivizumab despite being eligible. Three infants received palivizumab before hospitalization, none within the previous 30 days (eTable 3 in Supplement 1).

### Factors Associated With Risk for Intubation

**Figure 2.**
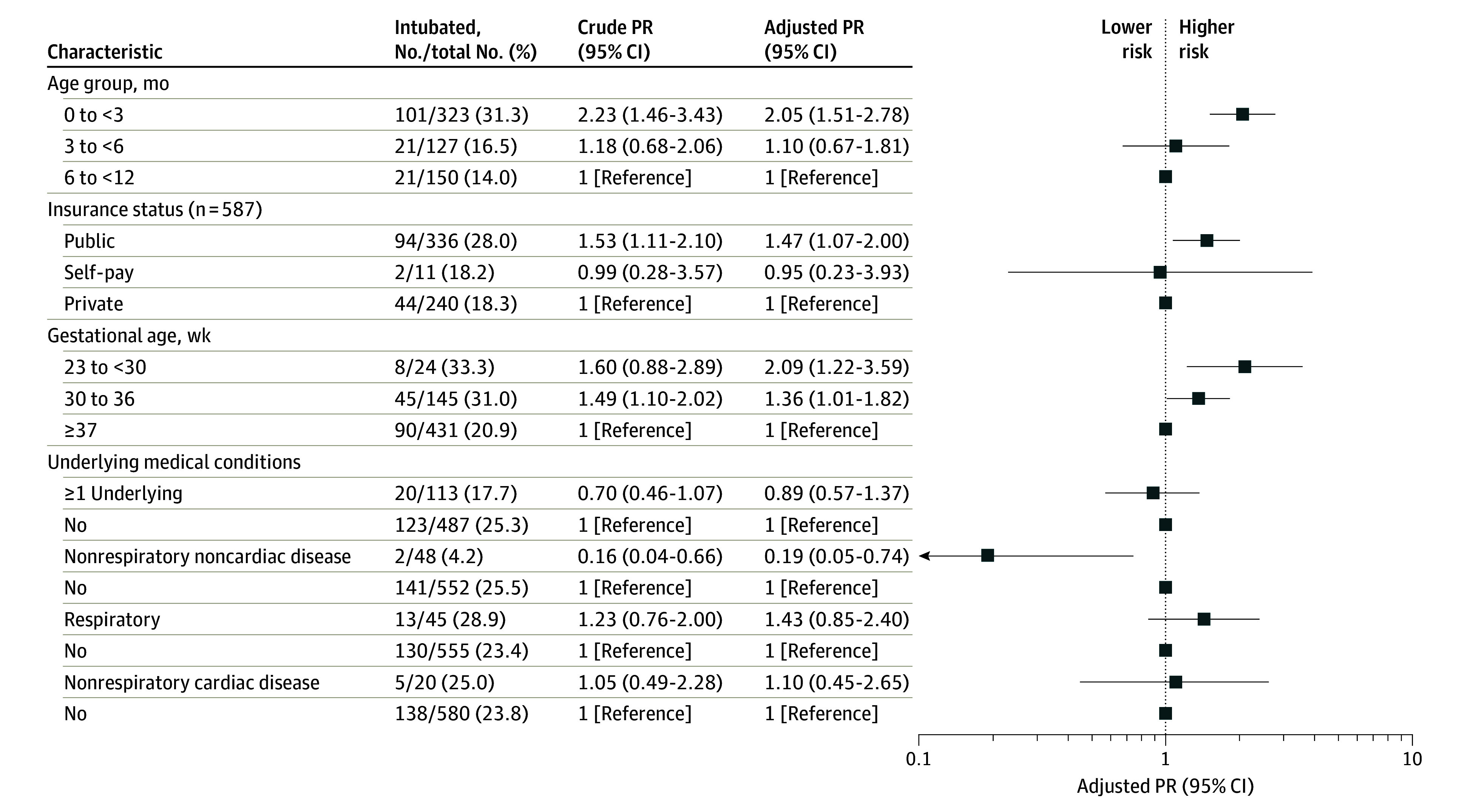
Factors Associated With Risk for Intubation in Infants Requiring Intensive Care for Respiratory Syncytial Virus Infection PR indicates prevalence ratio.

After adjusting for other factors, the risk of intubation was highest among infants younger than 3 vs 6 to 11 months old, those born prematurely (<37 weeks), and infants with public insurance compared with private (Figure 2). Five of 8 infants born at less than 30 weeks’ gestational age (62.5%) had a history of bronchopulmonary dysplasia (BPD), as did 1 of 45 infants (2.2%) born between 30 and 36 weeks’ gestational age. Although history of BPD was also associated with higher risk of intubation (adjusted prevalence ratio [aPR], 2.2; 95% CI, 1.0-4.8), this association is likely because BPD occurs exclusively in premature infants.

To determine if intubation risk was associated with high-flow nasal cannula use outside of ICU or high acuity units, we performed a post hoc analysis. During the study period, HFNC was administered in the wards of some hospitals (26 of 39 [67%]). The median proportion of patients intubated in the registry did not differ between hospitals who did and did not administer HFNC in the wards (25% vs 20%, respectively; *P* = .93; *t*_17.89 _= 0.08).

### Bacterial Coinfection and Viral Codetection

Within 3 days of admission, 42 of 143 intubated infants (29.4%) had an endotracheal culture performed, of which 28 of 42 (66.7%) had a tracheal or lower respiratory tract bacterial coinfection; 8 of 22 infants (36.4%) tested had a urinary tract infection; the frequency of testing and positivity was low for meningitis (0 of 1) and bacteremia (2 of 11 [18.2%]) (eTable 4 in Supplement 1). The most common coinfections detected in endotracheal specimens were nontypeable *Haemophilus influenzae* (12 of 28 [42.9%]), *Moraxella catarrhalis* (9 of 28 [32.1%]), and *Streptococcus pneumoniae* (8 of 28 [28.6%]). The urinary tract infections were with *E coli* and *Klebsiella spp* and the bloodstream infections were *Streptococcus pneumoniae* and methicillin-sensitive *Staphylococcus aureus*.

A total of 128 children (21.3%) tested positive for at least 1 non-RSV respiratory virus. Of 556 and 554 infants who underwent SARS-CoV-2 and influenza testing (Table 3), 19 (3.4%) and 7 (1.2%) had positive results, respectively. Among the 304 infants for whom a respiratory virus panel was performed, rhinovirus/enterovirus (RV/EV) was most often detected (85 infants [28.0%]) (eTable 5 in Supplement 1).

**Table 3.** Viral Codetection and Bacterial Coinfection Among Infants Admitted to the Intensive Care Unit or High Acuity Unit With Respiratory Syncytial Virus Infection

Codetection or coinfection	Infants, No. (%)		
	All (N = 600)	Nonintubated (n = 457)	Intubated (n = 143)
Viral codetection^a^			
SARS-CoV-2 tested	556 (92.7)	428 (93.7)	128 (89.5)
SARS-CoV-2 detected	19 (3.4)	16 (3.7)	3 (2.3)
Influenza tested	554 (92.3)	423 (92.6)	131 (91.6)
Influenza detected	7 (1.2)	6 (1.4)	1 (0.8)
Respiratory pathogen panel performed^b^	304 (50.7)	221 (48.4)	83 (58.0)
Rhinovirus/enterovirus detected	85 (28.0)	62 (28.1)	23 (27.7)
Any other virus positive	33 (10.9)	24 (10.9)	9 (10.8)
Probable or confirmed bacterial coinfection^a^			
Tracheal or lower respiratory tract	28 of 42 (66.7)	NA	28 of 42 (66.7)
Blood	2 of 11 (18.2)	0 of 6	2 of 5 (40.0)
Urine	8 of 22 (36.4)	5 of 13 (38.5)	3 of 9 (33.3)

Abbreviation: NA, not applicable.

^a^ Viral codetections from symptom onset up to 72 hours of admission and bacterial coinfections up to 3 days of hospital admission.

^b^ Results from other respiratory virus testing, including from respiratory pathogen panels, were collected where available. Other respiratory viruses included parainfluenza (types 1, 2, 3, and 4), human metapneumovirus, adenovirus, rhinovirus/enterovirus, and seasonal coronaviruses (NL63, OC43, HKU1, and 229E).

## Discussion

Our RSV-PIC registry included a nationally representative cohort of 600 RSV-confirmed infants admitted for intensive care in 39 hospitals across 27 US states during the peak 2022 RSV season. Almost one-quarter of the infants received invasive mechanical ventilation. Young infants aged less than 3 months had more severe clinical presentations and outcomes, including apnea at presentation, receipt of invasive mechanical ventilation, and longer ICU and hospital length of stays. Although having an underlying medical condition or history of prematurity is a factor associated with risk for severe RSV illness, most infants receiving ICU-level care were previously healthy and born at term. Younger and premature infants were at higher risk for intubation, but most intubated infants were born at term. Although mortality was rare, these findings emphasize the significant morbidity caused by RSV in US infants.

These infants with RSV-associated LRTI who required intermediate or intensive care during the so-called triple-demic of RSV, influenza, and SARS-CoV-2 in fall 2022 overwhelmed pediatric units across the US leading to widespread institutional ICU bed capacity challenges.^28^ Before the COVID-19 pandemic, the seasonality of RSV infections in temperate climates was consistent year-to-year, with cases increasing in late autumn, peaking in winter, and declining in early spring.^29^ However, nonpharmaceutical interventions, such as physical distancing and masking, introduced during the COVID-19 pandemic altered the circulation of RSV and other viruses, resulting in negligible RSV transmission during the 2020 to 2021 winter season.^30^ Simulation modeling predicted that out-of-season RSV outbreaks in 2021 and 2022 would be more intense than prepandemic outbreaks due to the expansion of the susceptible population.^31^ In the summer of 2021, RSV resurged out-of-season^32^ with reports of a markedly higher percentage of children admitted to the ICU.^33^ In 2022, RSV infections peaked again in October during the period where we captured data, with overall US incidence rates of RSV-associated hospitalizations roughly twice as high as at any prepandemic peak.^19^ In response to the RSV surge, the American Academy of Pediatrics and Children’s Hospital Association called for an emergency declaration on November 14, 2022, and the CDC issued a Health Alert Network Health Advisory.^34^

Although we enrolled infants aged up to 1 year, most were younger than 3 months, and this age group accounted for over 70% of intubated infants, most of whom were previously healthy. These results are consistent with previous studies in other high-resource countries. In a study of 604 Australian children (median age 4 months) with community-acquired RSV infections admitted to the ICU from 2005 to 2015, 55% of infants had no underlying medical condition and 32% required mechanical ventilation; 94% were admitted because of LRTI with a median duration of ICU stay of 3.7 days.^9^ In the Netherlands, of 2161 children younger than 2 years with RSV bronchiolitis from 2003 to 2016, 78% were younger than 3 months and 54% were term, but the percentage receiving invasive mechanical ventilation was higher at 72% and the median length of ICU stay was 8 days.^35^ Our cohort is similar to that from the US Pediatric Health Information Systems database from 2010 to 2019 in which 17% of children with bronchiolitis admitted to the ICU required invasive mechanical ventilation, with a median length of ICU stay of 2 to 3 days.^6^ Even though our registry included only infants with RSV, it is the most common cause of bronchiolitis.^36^ These studies highlight the need for prevention measures against severe RSV disease, including in young, healthy infants. In addition to preventing hospitalization associated with acute illness, preventing RSV LRTI may also change long-term outcomes potentially associated with RSV, including increased risk of developing recurrent wheezing/asthma or premature adult death.^37,38^

RSV is frequently codetected with other respiratory viruses, and codetection frequency depends on various factors, including age, setting, and type and timing of testing.^39^ In a previous study, we found that SARS-CoV-2 and influenza were associated with critical illness in children.^40^ However, in this cohort, neither virus was commonly detected, underscoring the role of RSV as a significant cause of severe respiratory disease in infants.^28^ Among the subset of infants who underwent respiratory pathogen panel testing, we found that RSV was most commonly codetected with RV/EV, as has been shown consistently in previous studies.^39,41^ However, the clinical significance of RV/EV codetection is unclear. In 1 study,^42^ clinical characteristics and outcomes of children younger than 2 years with RSV/RV codetection were largely similar to those with RSV-only detection but distinct from those with RV-only detection, which suggests a bystander role for RV when codetected with RSV. The frequency of serious bacterial infections in our cohort was fairly low. Of intubated infants that had a bacterial endotracheal culture sent within the first 3 hospitalization days, nearly two-thirds had pathogens considered true or probable infections. However, these infants comprised less than 5% of the entire RSV-PIC cohort. The role of bacterial coinfection in RSV severity remains unclear, and further investigation is needed.

### Limitations

Our findings are subject to limitations. Our cohort represents only the first 15 to 20 consecutive RSV cases at each hospital as we aimed to get a representative sampling across the US, limiting the influence of single centers or regions. Thus, we did not include all cases of severe RSV admitted to the ICU during the 2-month study period, which also did not encompass the full RSV season at these centers. In addition, including only clinician-ordered, laboratory-confirmed RSV cases may have resulted in missing cases that were not tested for RSV. Furthermore, although most infants were tested for influenza and SARS-CoV-2, only half were tested with a respiratory viral panel so we are unable to systematically assess the influence of viral codetection on disease severity. Not all intubated infants were tested for bacterial coinfection and it is not possible to acquire bacterial lower respiratory samples in nonintubated patients, so the role of bacterial coinfection may be underestimated. Moreover, the proportion of intubated infants varied across regions. Although we may have selected a more severely ill cohort of infants from the hospitals that administered HFNC in the wards, our post hoc analysis did not support this hypothesis. Thus, further investigation is essential to clarify regional disparities, which could involve differences in referral practices. The study period missed the RSV peak in some states, such as Florida, precluding meeting target enrollment for that site. Infants in our cohort may not be representative of all states, which may limit generalizability. Furthermore, since we relied on medical record abstractions, data missingness is possible. We also excluded infants that were admitted to the ICU for less than 24 hours and our study is biased toward the most severe RSV infections among infants.

## Conclusions

In conclusion, this surveillance registry of infants with critical RSV illness highlights that RSV causes significant morbidity in previously healthy term infants as well as those born prematurely and those with underlying conditions. Thus, prevention strategies are needed for all infants. In our study, only 2 of 17 infants eligible for palivizumab because of gestational age less than 29 weeks had documentation of receipt, highlighting potential barriers to administration and emphasizing the need to ensure that all eligible patients receive it in a timely manner. The recent experience during the COVID-19 pandemic has shown that community mitigation measures are effective in decreasing the circulation of non–COVID-19 respiratory viral pathogens, including RSV. The resurgence of RSV when these measures were lifted suggests that studies of nonpharmaceutical interventions may be important for future surges, but also underscores the need for therapeutic interventions to target all infants to reduce the overall burden of severe RSV disease. The clinical severity information in this investigation can also help guide the design of upcoming RSV prophylactic and maternal RSV vaccine effectiveness studies.

## Notes

Supplement 1. **eAppendix.** Definitions **eFigure.** Age Distribution of Infants Requiring Intensive Care for RSV Infection **eTable 1. **Underlying Medical Conditions Present in at Least 5 Critically Ill Infants With RSV, by Intubation Status **eTable 2. **Clinical Characteristics of Infants Who Received ECMO or Died During Their Hospitalization **eTable 3. **Clinical Characteristics of Infants Who Received Palivizumab Before Hospitalization **eTable 4. **Probable or Confirmed Bacterial Coinfections Isolated Within 72 Hours of Admission Among Critically Ill Children With RSV, by Intubation Status, Underlying Medical Condition Status, and Specimen Type **eTable 5. **Viral Codetections Among Critically Ill Children With RSV, by Intubation Status

Supplement 2.
Nonauthor Collaborators

Supplement 3.
Data Sharing Statement
