# Errors in Figure 2, Results, and Tables 1 and 2

**DOI:** 10.1001/jamanetworkopen.2024.28669

**Published:** 2024-07-25

In the Original Investigation titled “Infants Admitted to US Intensive Care Units for RSV Infection During the 2022 Seasonal Peak,”^1^ published August 15, 2023, the following corrections were made. First, the number of nonintubated infants 0 to 2 months old was corrected from 221 to 222 in Table 1. Second, infants from 1 site in the Northeast were misclassified as being from the South; these numbers were corrected in the Results text and Table 1. Third, the number of infants on extracorporeal membrane oxygenation was corrected from 5 to 4 infants in Table 2. Fourth, text was updated to clarify that the gestational age in weeks category cutoff was less than 30 weeks and 30 to 36 weeks in the Results text and Figure 2. Lastly, the classification of 1 infant who received continuous positive airway pressure instead of high-flow nasal cannula as the highest level of respiratory support in the first 24 hours was corrected in Table 2, and the data source for these interventions was clarified with a footnote. Two footnotes were added in Table 1 to clarify that the percent with prematurity was only for those infants with documented prematurity status and that multiple pregnancy status was collected only for infants less than 90 days of age. The original footnote b in Table 2 for median days ventilated was removed because the denominator is complete. This article has been corrected.^1^
