# Supplementary material for: Infants Admitted to US Intensive Care Units for RSV Infection During the 2022 Seasonal Peak
Source: JAMA Netw Open. 2023 Aug 15;6(8):e2328950. doi: 10.1001/jamanetworkopen.2023.28950 (PMC10427947; doi:10.1001/jamanetworkopen.2023.28950)
Supplement: Supplement 1. — eAppendix. Definitions eFigure. Age Distribution of Infants Requiring Intensive Care for RSV Infection eTable 1. Underlying Medical Conditions Present in at Least 5 Critically Ill Infants With RSV, by Intubation Status eTable 2. Clinical Characteristics of Infants Who Received ECMO or Died During Their Hospitalization eTable 3. Clinical Characteristics of Infants Who Received Palivizumab Before Hospitalization eTable 4. Probable or Confirmed Bacterial Coinfections Isolated Within 72 Hours of Admission Among Critically Ill Children With RSV, by Intubation Status, Underlying Medical Condition Status, and Specimen Type eTable 5. Viral Codetections Among Critically Ill Children With RSV, by Intubation Status [file jamanetwopen-e2328950-s001.pdf]

Supplemental Online Content

Halasa N, Zambrano LD, Amarin JZ, et al; RSV-PIC Investigators. Infants admitted to US intensive care units for RSV infection during the 2022 seasonal peak. *JAMA Netw Open*. 2023;6(8):e2328950. doi:10.1001/jamanetworkopen.2023.28950

eAppendix. Definitions

eFigure. Age Distribution of Infants Requiring Intensive Care for RSV Infection

eTable 1. Underlying Medical Conditions Present in at Least 5 Critically Ill Infants With RSV, by Intubation Status

eTable 2. Clinical Characteristics of Infants Who Received ECMO or Died During Their Hospitalization

eTable 3. Clinical Characteristics of Infants Who Received Palivizumab Before Hospitalization

eTable 4. Probable or Confirmed Bacterial Coinfections Isolated Within 72 Hours of Admission Among Critically Ill Children With RSV, by Intubation Status, Underlying Medical Condition Status, and Specimen Type

eTable 5. Viral Codetections Among Critically Ill Children With RSV, by Intubation Status

This supplemental material has been provided by the authors to give readers additional information about their work.

© 2023 Halasa N et al. *JAMA Network Open.*

eAppendix. Definitions

Underlying Medical Conditions

Underlying conditions included respiratory (chronic restrictive lung disease, bronchopulmonary dysplasia, cystic fibrosis, recurrent aspiration into lungs, pulmonary hypertension, interstitial lung disease, agenesis, hypoplasia, or other congenital lung deformity, obstructive sleep apnea, tracheomalacia/bronchomalacia/laryngomalacia, subglottic or supraglottic stenosis, tracheoesophageal fistula, tracheal vascular ring, micrognathia/retrognathia, choanal atresia), cardiac (congenital heart disease, cardiac repair, acquired heart disease, arrhythmia, systemic hypertension), neurologic/neuromuscular, oncologic, immunosuppressive, rheumatologic (autoimmune) disorder, hematologic, renal or urologic, gastrointestinal or hepatic, endocrine, metabolic or genetic disorders, and atopic/allergic conditions. Non-respiratory, non-cardiac disease included all those with an underlying medical condition that was *not* respiratory or cardiac in nature. Non-respiratory cardiac diseases included those with an underlying cardiac condition without a co-occurring respiratory condition.

Pediatric Acute Respiratory Distress Syndrome (PARDS)

PARDS was defined by the following criteria being met within 24 hours: acute onset of hypoxemia; chest imaging with new infiltrates; respiratory failure not explained by cardiac failure or volume overload; and either P_aO_2/F_iO_2 ratio $\leq$300 or, if arterial blood gas was not available, SpO_2/F_iO_2 <264 (if SpO_2 <97) if on continuous positive airway pressure (CPAP) >5 cm H_2O, bilevel positive airway pressure (BiPAP), or invasive mechanical ventilation.

© 2023 Halasa N et al. *JAMA Network Open.*

eFigure. Age Distribution of Infants Requiring Intensive Care for RSV Infection Distribution of age (in months) among 600 infants admitted to the intensive care unit or high acuity unit with respiratory syncytial virus infection, stratified by intubation status.

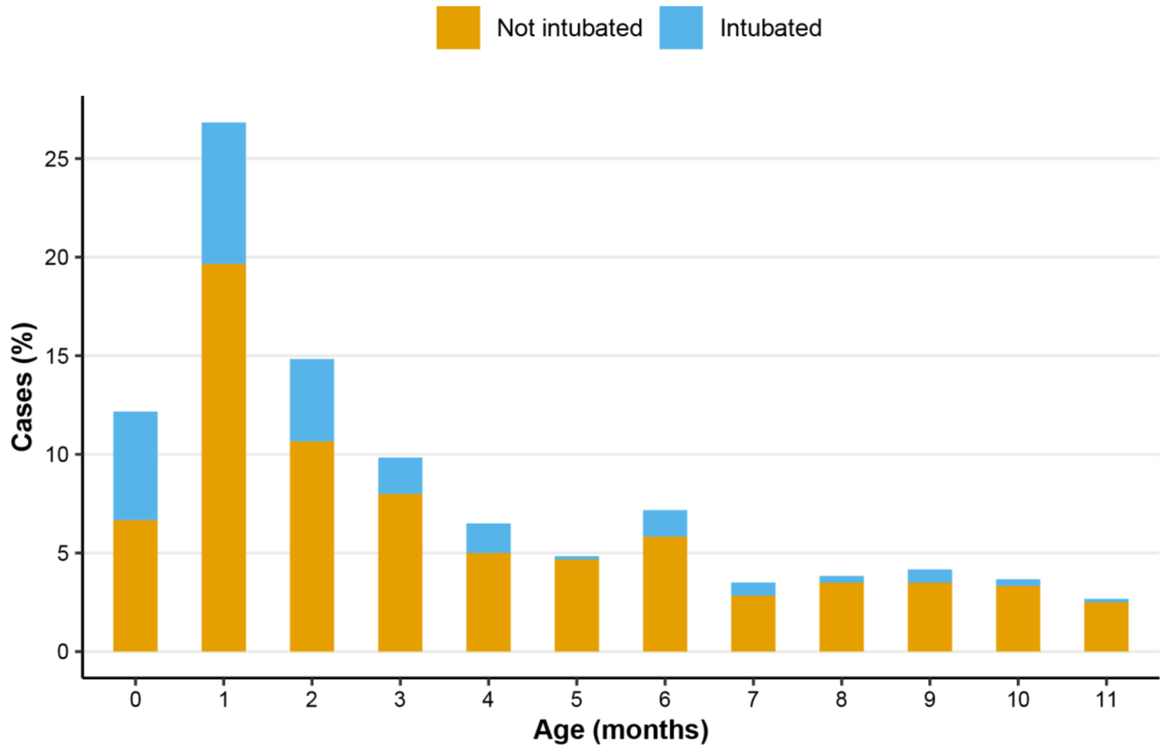

© 2023 Halasa N et al. *JAMA Network Open.*

eTable 1. Underlying Medical Conditions Present in at Least 5 Critically Ill Infants With RSV, by Intubation Status

Underlying medical condition	All (*n*=600)	Non-intubated (*n*=457)	Intubated (*n*=143)	*P* value
Respiratory disorder	45 (7.5)	32 (7.0)	13 (9.1)	0.41
Reactive airway disease/recurrent wheezing	8 (1.3)	8 (1.8)	0	0.21*
Chronic lung disease	22 (3.7)	14 (3.1)	8 (5.6)	0.16
Bronchopulmonary dysplasia	19 (3.2)	13 (2.8)	6 (4.2)	0.42
Upper airway disorder	12 (2.0)	9 (2.0)	3 (2.1)	>0.99 *
Tracheomalacia, bronchomalacia, or laryngomalacia	7 (1.2)	5 (1.1)	2 (1.4)	0.67*
Cardiovascular disorder	35 (5.8)	24 (5.3)	11 (7.7)	0.28
Congenital heart disease	31 (5.2)	21 (4.6)	10 (7.0)	0.26
Atrial septal defect, ventricular septal defect, atrioventricular septal defect, or atrioventricular canal	20 (3.3)	14 (3.1)	6 (4.2)	0.51
Abnormalities of aortic arch (e.g., coarctation of aorta, double aortic arch, interrupted aortic arch, hypoplastic aortic arch, right-sided aortic arch, truncus arteriosus)	6 (1.0)	3 (0.7)	3 (2.1)	0.15*
History of cardiac repair (surgery or interventional catheterization)	11 (1.8)	7 (1.5)	4 (2.8)	0.30*
Neurologic or neuromuscular disorder	13 (2.2)	9 (2.0)	4 (2.8)	0.52*
Disorders of tone (e.g., hypotonia, dystonia, spasticity, hypertonia, cerebral palsy, spastic quadriplegia, quadriparesis)	5 (0.8)	3 (0.7)	2 (1.4)	0.34*
Intraventricular hemorrhage, anoxic or traumatic brain injury, hypoxic ischemic encephalopathy, or static encephalopathy	5 (0.8)	5 (1.1)	0	0.60*
Hematologic disorder	5 (0.8)	4 (0.9)	1 (0.7)	>0.99 *
Renal or urologic dysfunction	13 (2.2)	11 (2.4)	2 (1.4)	0.74*
Gastrointestinal or hepatic disorder	35 (5.8)	27 (5.9)	8 (5.6)	0.89
Gastroesophageal reflux requiring treatment (e.g. surgery or medication)	22 (3.7)	18 (3.9)	4 (2.8)	0.62*

© 2023 Halasa N et al. *JAMA Network Open.*

Underlying medical condition	All (*n*=600)	Non-intubated (*n*=457)	Intubated (*n*=143)	*P* value
Feeding through a tube (i.e. not a temporary nasogastric tube but a long-lasting gastric or jejunal tube)	15 (2.5)	9 (2.0)	6 (4.2)	0.14
Dysphagia (or swallowing dysfunction)	7 (1.2)	5 (1.1)	2 (1.4)	0.67*
Surgical or congenital gastrointestinal disease (e.g., short gut or bowel syndrome, gastroschisis, omphalocele, malrotation, midgut volvulus, Hirschsprung disease)	5 (0.8)	4 (0.9)	1 (0.7)	>0.99*
Endocrine disorder	7 (1.2)	6 (1.3)	1 (0.7)	>0.99*
Hypothyroidism	5 (0.8)	4 (0.9)	1 (0.7)	>0.99*
Metabolic or genetic disorder	13 (2.2)	11 (2.4)	2 (1.4)	0.74*
Trisomy 21	8 (1.3)	8 (1.8)	0	0.21*
Atopic or allergic condition	11 (1.8)	11 (2.4)	0	0.07*
Eczema (or atopic dermatitis)	10 (1.7)	10 (2.2)	0	0.13*

*Indicates that fisher exact test was run since the cell sizes were small (<5).

© 2023 Halasa N et al. *JAMA Network Open.*

eTable 2. Clinical Characteristics of Infants Who Received ECMO or Died During Their Hospitalization

For privacy, age is rounded to the closest month and gestational age is given in a range.

**Characteristic**	**Death 1 (ECMO)**	**Death 2 (no ECMO)**	**ECMO 1 Survivor**	**ECMO 2 Survivor**	**ECMO 3 Survivor**
Age (months)	10	8	11	2	1
Sex	Male	Female	Male	Male	Male
Race	Unknown	Unknown	Black	White	Black
Ethnicity	Hispanic/Latino	Unknown	Non-Hispanic	Non-Hispanic	Non-Hispanic
Region	South	South	Midwest	Midwest	South
Gestational age at birth (weeks)	27–28	≥37	<25	29–30	≥37
Underlying conditions	BPD; failure to thrive	None	BPD	None	None
Symptoms prior to admission	Cough, runny nose, rapid or shallow breathing, wheezing, chest retractions or abdominal breathing, poor feeding, nasal flaring	Fever, cough, rapid or shallow breathing, poor feeding, difficult to arouse or increased sleepiness, seizure	Fever, cough, runny nose, rapid or shallow breathing, wheezing, chest retractions or abdominal breathing, irritability	Cough, runny nose, apnea, poor feeding, irritability, difficult to arouse or increased sleepiness	Cough, runny nose, rapid or shallow breathing, apnea, poor feeding, sleepiness/difficult to rouse, pale, grey, and mottled skin with capillary refill > 3 seconds
Day of death	18 days	7 days	N/A	N/A	N/A
Invasive mechanical ventilation	Yes	Yes	Yes	Yes	Yes

© 2023 Halasa N et al. *JAMA Network Open.*

Characteristic	Death 1 (ECMO)	Death 2 (no ECMO)	ECMO 1 Survivor	ECMO 2 Survivor	ECMO 3 Survivor
Bacterial lower respiratory tract coinfection (endotracheal tube or tracheostomy or bronchoscopy)[a]	· *Moraxella catarrhalis*; abundance= abundant (day 8)<br>· *Staphylococcus aureus* (methicillin-resistant); abundance= moderate (day 8)<br>· *Serratia marcescens* abundance=moderate (day 17)	· *Pseudomonas*; abundance= abundant (day 6)	· *Haemophilus influenzae* (non-typeable); abundance= many (day 1=day of admission)<br>· *Staphylococcus aureus* (methicillin-sensitive); abundance=few (day 1)<br>· *Moraxella catarrhalis*; abundance= moderate (day 1)<br>· Viridans group Streptococci; abundance= many (day 1)<br>· *Enterobacter cloacae*; abundance=few (day 16)	None	· *Pseudomonas*; abundance=few (day 19)<br>· *Stenotrophomonas maltophilia*; abundance=few (day 19)
Viral codetection[a]	Rhinovirus/enterovirus	Coronavirus OC43	Rhinovirus/enterovirus	Influenza (untyped) codetection; other viral tests not performed	Adenovirus

[a]Bacterial coinfection and viral codetection were within 72 hours of admission. **Abbreviations:** ECMO, extracorporeal membrane oxygenation; BPD, bronchopulmonary dysplasia.

© 2023 Halasa N et al. *JAMA Network Open.*

eTable 3. Clinical Characteristics of Infants Who Received Palivizumab Before Hospitalization

For privacy, age is rounded to the closest month, and gestational age is given in a range.

**Characteristic**	**Palivizumab Infant 1 (Death 1 from eTable 2)**	**Palivizumab Infant 2**	**Palivizumab Infant 3**
Age (months)	10	8	10
Sex	Male	Male	Male
Race	Unknown	White	White
Ethnicity	Hispanic/Latino	Non-Hispanic	Non-Hispanic
Region	South	South	Northeast
Palivizumab received within prior 30 days?	No	No	No
Gestational age at birth (weeks)	27-28	28–29	≥37
Underlying conditions	BPD; Failure to thrive	Congenital heart defect; BPD; Hypothyroidism	Trisomy 21; Congenital heart defect; Tracheobronchomalacia; Gastroesophageal reflux with feeding tube; Hypothyroidism
Symptoms prior to admission	Cough, runny nose, rapid or shallow breathing, wheezing, chest retractions or abdominal breathing, poor feeding, nasal flaring	Cough, runny nose, rapid or shallow breathing, wheezing, chest retractions or abdominal breathing	Fever, cough, runny nose, rapid or shallow breathing, wheezing, chest retractions or abdominal breathing, diarrhea, poor feeding, irritability
Invasive mechanical ventilation	Yes	Yes	No
ECMO	Yes	No	No
Bacterial coinfection documented	*Moraxella catarrhalis* (LRT, abundant); MRSA (LRT, moderate); *S. marcescens* (LRT, moderate)	No	No
Viral codetection documented	Rhinovirus/enterovirus	No	No

Abbreviations: BPD, bronchopulmonary dysplasia; ECMO, extracorporeal membrane oxygenation.

© 2023 Halasa N et al. *JAMA Network Open.*

eTable 4. Probable or Confirmed Bacterial Coinfections Isolated Within 72 Hours of Admission Among Critically Ill Children With RSV, by Intubation Status, Underlying Medical Condition Status, and Specimen Type

	**All (*n*=600)**	**Non-intubated (*n*=457)**	**Intubated (*n*=143)**	**History of ≥1 underlying condition (*n*=113)**
Tracheal/lower respiratory pathogen	28/42 (66.7)	NA	28/42 (66.7)	2/6 (33.3)
Streptococcus pneumoniae	8/28 (28.6)	NA	8/28 (28.6)	0
Moraxella catarrhalis	9/28 (32.1)	NA	9/28 (32.1)	3/6 (50.0)
Haemophilus influenzae non-typeable	12/28 (42.9)	NA	12/28 (42.9)	2/6 (33.3)
Haemophilus influenzae type B	1/28 (3.6)	NA	1/28 (3.6)	0
Pseudomonas spp.	1/28 (3.6)	NA	1/28 (3.6)	1/6 (16.7)
Methicillin-resistant *Staphylococcus aureus*	1/28 (3.6)	NA	1/28 (3.6)	0
Methicillin-sensitive *Staphylococcus aureus*	1/28 (3.6)	NA	1/28 (3.6)	1/6 (16.7)
Escherichia coli	1/28 (3.6)	NA	1/28 (3.6)	0
Klebsiella spp.	1/28 (3.6)	NA	1/28 (3.6)	0
Blood	2/11 (18.2)	0/6 (0)	2/5 (40.0)	0
Streptococcus pneumoniae	1/2 (50.0)	0	1/2 (50.0)	0
Methicillin-sensitive *Staphylococcus aureus*	1/2 (50.0)	0	1/2 (50.0)	0
Acinetobacter baumannii	1/2 (50.0)	0	1/2 (50.0)	0
Urine	8/22 (36.4)	5/13 (38.5)	3/9 (33.3)	1/4 (25.0)
Escherichia coli	5/8 (62.5)	3/5 (60.0)	2/3 (66.7)	1 (100)
Klebsiella spp.	3/8 (37.5)	2/5 (40.0)	1/3 (33.3)	0

© 2023 Halasa N et al. *JAMA Network Open.*

eTable 5. Viral Codetections Among Critically Ill Children With RSV, by Intubation Status

	**All (*n*=600)**	**Non-intubated (*n*=457)**	**Intubated (*n*=143)**
Respiratory viral panel performed	304	221	83
Rhinovirus/enterovirus detected	85 (28.0)	62 (28.1)	23 (27.7)
All other virus positive	33 (10.9)	24 (10.9)	9 (10.8)
Parainfluenza virus	13 (4.3)	9 (4.1)	4 (4.8)
Type 1	8 (2.6)	5 (2.3)	3 (3.6)
Type 2	3 (1.0)	2 (0.9)	1 (1.2)
Type 3	4 (1.3)	3 (1.4)	1 (1.2)
Type 4	6 (2.0)	4 (1.8)	2 (2.4)
Human metapneumovirus	5 (1.6)	4 (1.8)	1 (1.2)
Adenovirus	17 (5.6)	13 (5.9)	4 (4.8)
Seasonal coronaviruses	5 (1.6)	2 (0.9)	3 (3.6)
229E	1 (0.3)	0	1 (1.2)
HKU1	2 (0.7)	0	2 (2.4)
NL63	2 (0.7)	1 (0.5)	1 (1.2)
OC43	3 (1.0)	1 (0.5)	2 (2.4)

© 2023 Halasa N et al. *JAMA Network Open.*
