# Supplementary material for: Infants Admitted to US Intensive Care Units for RSV Infection During the 2022 Seasonal Peak
Source: JAMA Netw Open. 2023 Aug 15;6(8):e2328950. doi: 10.1001/jamanetworkopen.2023.28950 (PMC10427947; doi:10.1001/jamanetworkopen.2023.28950)
Supplement: Supplement 2. — Nonauthor Collaborators [file jamanetwopen-e2328950-s002.pdf]

Supplemental Online Content: Nonauthor Collaborators
*First name, last name, and suffix (if applicable) are required and will appear in PubMed.

*Group Name(s): RSV-PIC Investigators							
*First Name and Middle Initial(s)	*Last Name	*Suffix (eg, Jr, III)	Academic Degrees	Institution	Location (city, state/province, country)	Role or Contribution, eg, chair, principal investigator	Group (if more than 1 Group listed in the byline) and/or Subgroup (eg, Steering Committee)
Merry	Tomcany		BSN, RN	Akron Children's Hospital	Akron, Ohio, USA	Collaborator	
Kelly N.	Michelson		MD, MPH	Ann & Robert H. Lurie Children's Hos	Chicago, Illinois, USA	Collaborator	
Heather E.	Price		MS, CRA	Ann & Robert H. Lurie Children's Hos	Chicago, Illinois, USA	Collaborator	
Ronald C.	Sanders	Jr	MD	Arkansas Children's Hospital	Little Rock, Arkansas, USA	Collaborator	
Lexie	Dixon		BA	Arkansas Children's Hospital	Little Rock, Arkansas, USA	Collaborator	
Katri V.	Typpo		MD, MPH	Banner Children's at Diamond Childre	Tucson, Arizona, USA	Collaborator	
Ilana	Harwayne-Gidansky		MD, MA	Bernard & Millie Duker Children's Ho	Albany, New York, USA	Collaborator	
Suden	Kucukak		MD	Boston Children's Hospital	Boston, Massachusetts, US	Collaborator	
Elizabeth R.	McNamara		BSN, RN	Boston Children's Hospital	Boston, Massachusetts, US	Collaborator	
Sabrina R.	Chen		BS	Boston Children's Hospital	Boston, Massachusetts, US	Collaborator	
Eve	Listerud			Boston Children's Hospital	Boston, Massachusetts, US	Collaborator	
Ofelia	Vargas-Shiraishi		BS	Children's Health of Orange County	Orange, California, USA	Collaborator	
Betty	Oberle		BSN, RN	Children's Hospital & Medical Center	Omaha, Nebraska, USA	Collaborator	
Frances	Zorensky		BA	Children's Hospital Colorado	Aurora, Colorado, USA	Collaborator	
Rachel	Mansour		BSN, RN, Cl	Children's Hospital Colorado	Aurora, Colorado, USA	Collaborator	
Jaycee	Jumarang		MD	Children's Hospital Los Angeles	Los Angeles, California, US/	Collaborator	
Marla	Johnston		MSN, RN	Children's Hospital New Orleans	New Orleans, Louisiana, US	Collaborator	
Jenny L.	Bush		RNC, BSN	Children's Hospital of Philadelphia	Philadelphia, Pennsylvania,	Collaborator	
Shawn	Dickey		MHA	Children's Hospital of Philadelphia	Philadelphia, Pennsylvania,	Collaborator	
Shannon	Hill		BSN	Children's Mercy Kansas City	Kansas City, Missouri, USA	Collaborator	
Melissa	Sullivan		BSN	Children's Mercy Kansas City	Kansas City, Missouri, USA	Collaborator	
Abigail	Kietzman		ACRP-CP	Children's Mercy Kansas City	Kansas City, Missouri, USA	Collaborator	
Candice	Colston			Children's of Alabama	Birmingham, Alabama, US/	Collaborator	
Meghan	Murdock		RN	Children's of Alabama	Birmingham, Alabama, US/	Collaborator	
Heather	Kelley		RN	Children's of Alabama	Birmingham, Alabama, US/	Collaborator	
Laura	Wright-Sexton		MD	Children's of Mississippi	Jackson, Mississippi, USA	Collaborator	
Maygan	Martin		RN	Children's of Mississippi	Jackson, Mississippi, USA	Collaborator	
Lora	Martin		MSN	Children's of Mississippi	Jackson, Mississippi, USA	Collaborator	
Lacy	Malloch		BS	Children's of Mississippi	Jackson, Mississippi, USA	Collaborator	
Kayla	Patterson		MS	Children's of Mississippi	Jackson, Mississippi, USA	Collaborator	

Supplemental Online Content: Nonauthor Collaborators
*First name, last name, and suffix (if applicable) are required and will appear in PubMed.

***First Name and Middle Initial(s)**	***Last Name**	***Suffix (eg, Jr, III)**	Academic Degrees	Institution	Location (city, state/province, country)	Role or Contribution, eg, chair, principal investigator	Group (if more than 1 Group listed in the byline) and/or Subgroup (eg, Steering Committee)
Cameron	Sanders		BS	Children's of Mississippi	Jackson, Mississippi, USA	Collaborator	
Chelsea	Rohlfs		MBA	Cincinnati Children's Hospital Medica	Cincinnati, Ohio, USA	Collaborator	
Marilyn	Rice		MS	Cincinnati Children's Hospital Medica	Cincinnati, Ohio, USA	Collaborator	
Miranda	Howard		BS	Cincinnati Children's Hospital Medica	Cincinnati, Ohio, USA	Collaborator	
Makayla	Murphy		MPH	Connecticut Children's Medical Cente	Hartford, Connecticut, USA	Collaborator	
Vijaya L.	Soma		MD	Hassenfeld Children's Hospital at NYU	New York, New York, USA	Collaborator	
Adam J.	Ratner		MD	Hassenfeld Children's Hospital at NYU	New York, New York, USA	Collaborator	
Megan J.	Job		BA	Hassenfeld Children's Hospital at NYU	New York, New York, USA	Collaborator	
Colleen	Mennie		BSN	Johns Hopkins Children's Center	Baltimore, Maryland, USA	Collaborator	
Kamala	Simkhada		BSN, MPH	Johns Hopkins Children's Center	Baltimore, Maryland, USA	Collaborator	
Noelle M.	Drapeau		BA	Mayo Clinic Children's Center	Rochester, Minnesota, USA	Collaborator	
Supriya	Behl		MSc	Mayo Clinic Children's Center	Rochester, Minnesota, USA	Collaborator	
Kristina A.	Betters		MD	Monroe Carell Jr. Children's Hospital	Nashville, Tennessee, USA	Collaborator	
Haya	Hayek		MD	Monroe Carell Jr. Children's Hospital	Nashville, Tennessee, USA	Collaborator	
Molly	Maranto		BS	MUSC Shawn Jenkins Children's Hosp	Charleston, South Carolina,	Collaborator	
Aubrie	Waters		BS	MUSC Shawn Jenkins Children's Hosp	Charleston, South Carolina,	Collaborator	
Maggie	Flowers		BSN, RN	Nationwide Children's Hospital	Columbus, Ohio, USA	Collaborator	
Kevin	Havlin		MD	Norton Children's Hospital	Louisville, Kentucky, USA	Collaborator	
Jamie	Furlong-Dillard		DO	Norton Children's Hospital	Louisville, Kentucky, USA	Collaborator	
Melissa	Porter		MD	Norton Children's Hospital	Louisville, Kentucky, USA	Collaborator	
Jennifer	Nason		RN, BSN, C	Norton Children's Hospital	Louisville, Kentucky, USA	Collaborator	
Madison	Ray		RN	Norton Children's Hospital	Louisville, Kentucky, USA	Collaborator	
Kristen	Gossett		BS, CCRC	Norton Children's Hospital	Louisville, Kentucky, USA	Collaborator	
Hillary	Crandall		MD, PhD	Primary Children's Hospital	Salt Lake City, Utah, USA	Collaborator	
Evan	Heller		BS, CCRC	Primary Children's Hospital	Salt Lake City, Utah, USA	Collaborator	
Jennifer	Foley		RN, BSN	Rady Children's Hospital San Diego	San Diego, California, USA	Collaborator	
Rajashri	Rasal		MPH, CCRP	Rainbow Babies and Children's Hospi	Cleveland, Ohio, USA	Collaborator	
Christine	Marlow		BA	Rainbow Babies and Children's Hospi	Cleveland, Ohio, USA	Collaborator	
Anurithi	Senthil		BA	Rainbow Babies and Children's Hospi	Cleveland, Ohio, USA	Collaborator	
Kimberly	Myers		BSN	Rainbow Babies and Children's Hospi	Cleveland, Ohio, USA	Collaborator	
Betsy	Tudor		CCMA	Riley Hospital for Children	Indianapolis, Indiana, USA	Collaborator	
Amanda	Adler		BS	Seattle Children's Hospital	Seattle, Washington, USA	Collaborator	

Supplemental Online Content: Nonauthor Collaborators
*First name, last name, and suffix (if applicable) are required and will appear in PubMed.

***First Name and Middle Initial(s)**	***Last Name**	***Suffix (eg, Jr, III)**	Academic Degrees	Institution	Location (city, state/province, country)	Role or Contribution, eg, chair, principal investigator	Group (if more than 1 Group listed in the byline) and/or Subgroup (eg, Steering Committee)
Nereyda	Garcia		MD	Texas Children's Hospital	Houston, Texas, USA	Collaborator	
Natalie	Treister		BS	UCSF Benioff Children's Hospital Oakl	Oakland, California, USA	Collaborator	
Patrick S.	McQuillen		MD	UCSF Benioff Children's Hospital San	San Francisco, California, U	Collaborator	
Kathleen	Sun		BA	UCSF Benioff Children's Hospital San	San Francisco, California, U	Collaborator	
Denise	Villarreal-Chico		BA	UCSF Benioff Children's Hospital San	San Francisco, California, U	Collaborator	
Sophia	Kainaroi		BS	UPMC Children's Hospital of Pittsburg	Pittsburgh, Pennsylvania, U	Collaborator	
John V.	Williams		MD	UPMC Children's Hospital of Pittsburg	Pittsburgh, Pennsylvania, U	Collaborator	
