# Supplementary material for: Infants Admitted to US Intensive Care Units for RSV Infection During the 2022 Seasonal Peak
Source: JAMA Netw Open. 2023 Aug 15;6(8):e2328950. doi: 10.1001/jamanetworkopen.2023.28950 (PMC10427947; doi:10.1001/jamanetworkopen.2023.28950)
Supplement: Supplement 3. — Data Sharing Statement [file jamanetwopen-e2328950-s003.pdf]

Data Sharing Statement

Halasa. Infants Admitted to US Intensive Care Units for RSV Infection During the 2022 Seasonal Peak. *JAMA Netw Open*. Published August 15, 2023.
doi:10.1001/jamanetworkopen.2023.28950

Data
Data available: Yes
Data types: Deidentified participant data
How to access data: only upon formal request
When available: With publication

Supporting Documents
Document types: None

Additional Information
Who can access the data: researchers whose proposed use of the data has been approved
Types of analyses: For a specified purpose
Mechanisms of data availability: with a signed data access agreement
